# Predictive Value of ^18^F-Fluorodeoxyglucose Positron-Emission Tomography Metabolic and Volumetric Parameters for Systemic Metastasis in Tonsillar Cancer

**DOI:** 10.3390/cancers14246242

**Published:** 2022-12-18

**Authors:** Jooin Bang, Hye Lim Park, Ie Ryung Yoo, Hyun-Il Shin, Geun-Jeon Kim, Dong-Il Sun, Sang-Yeon Kim

**Affiliations:** 1 Department of Otolaryngology-Head and Neck Surgery, Seoul St. Mary’s Hospital, College of Medicine, The Catholic University of Korea, Seoul 06591, Republic of Korea; 2 Division of Nuclear Medicine, Department of Radiology, Eunpyeong St. Mary’s Hospital, College of Medicine, The Catholic University of Korea, Seoul 06591, Republic of Korea; 3 Division of Nuclear Medicine, Department of Radiology, Seoul St. Mary’s Hospital, College of Medicine, The Catholic University of Korea, Seoul 06591, Republic of Korea

**Keywords:** positron-emission tomography, tonsillar neoplasms, neoplasm metastasis, metabolic tumor volume, predictive value of tests

## Abstract

**Simple Summary:**

The need to predict failure of locoregional or systemic control in tonsillar cancer is increasing. Therefore, we explored whether several ^18^F-fluorodeoxyglucose (FDG)-related parameters can predict metastasis. We retrospectively reviewed the medical records of 55 patients with tonsil squamous cell carcinoma who underwent pretreatment ^18^F-FDG positron-emission tomography/computed tomography (PET/CT) followed by primary surgery. In our cohort, the metabolic tumor volume (MTV_2.5_), total lesion glycolysis, and tumor-to-liver uptake ratio were higher than those of the patients without systemic metastasis. The MTV_2.5_ value was significantly different between the groups even when the values for the primary tumor and metastatic lymph nodes were summed. Our results will aid prognostic predictions and individualized post-treatment surveillance; additional systemic therapy should be considered for patients at high risk of disease control failure.

**Abstract:**

Although the prognosis of tonsillar cancer (human papillomavirus-positive oropharyngeal squamous cell carcinoma) is improving, disease control failure (distant metastasis) still occurs in some cases. We explored whether several ^18^F-fluorodeoxyglucose (FDG) positron-emission tomography (PET) parameters can predict metastasis. We retrospectively reviewed the medical records of 55 patients with tonsil squamous cell carcinoma who underwent pretreatment ^18^F-FDG positron-emission tomography/computed tomography (PET/CT) followed by primary surgery. During the follow-up period, systemic metastases were found in 7 of the 55 patients. The most common sites were the lungs (33%), bone (22%), brain/skull base (22%), small bowel (11%), and liver (11%). Pathologically, P53 mutation was less common in patients with systemic metastasis (41.7% vs. 14.3%, *p* = 0.054) than without systemic metastasis. In terms of PET parameters, the metabolic tumor volume (MTV_2.5_) and total lesion glycolysis (TLG_2.5_) values were lower in the primary tumor, and higher in the metastatic lymph nodes, of human papillomavirus (HPV)-positive compared to HPV-negative patients (all *p* < 0.05). The MTV_2.5_, TLG_2.5_, and tumor–to–liver uptake ratio were 36.07 ± 54.24 cm^3^, 183.46 ± 298.62, and 4.90 ± 2.77, respectively, in the systemic metastasis group, respectively; all of these values were higher than those of the patients without systemic metastasis (all *p* < 0.05). The MTV_2.5_ value was significantly different between the groups even when the values for the primary tumor and metastatic lymph nodes were summed (53.53 ± 57.78 cm^3^, *p* = 0.036). The cut-off value, area under the curve (95% confidence interval), sensitivity, and specificity of MTV_2.5_ for predicting systemic metastasis were 11.250 cm^3^, 0.584 (0.036–0.832), 0.571, and 0.565, respectively. The MTV_2.5_ of metastatic lymph nodes and summed MTV_2.5_ values of the primary tumor and metastatic lymph nodes were significantly higher in tonsillar cancer patients with than without systemic metastases. We suggest PET/CT scanning for pre-treatment cancer work-up and post-treatment surveillance to consider additional systemic therapy in patients with a high risk of disease control failure.

## 1. Introduction

Smoking and alcohol have been recognized as traditional triggers for developing tumorous conditions. In recent years, however, the oropharyngeal cancer has been spotlighted as its prevalence and prognosis due to novel discoveries in pathophysiology over the past two decades [1,2]. Although the survival rate of patients with human papillomavirus (HPV)-positive tonsillar cancer is increasing, some patients have a dire prognosis due to systemic metastasis reflecting disease control failure [3,4]. Thus, many studies have aimed to predict and control locoregional or distant metastases [5,6].

Nodal status at diagnosis has a major impact on disease control. Vainshtein et al. reported that matted metastatic nodes strongly predicted a poor prognosis, because they increase the risk of distant metastasis [7]. Additionally, the nodal burden (number or volume of metastatic nodes) was independently associated with mortality from head and neck cancer, and was significantly more important than traditional pathological features such as extranodal extension or a positive resection margin [8,9].

Given the increasing proportion of HPV-positive tonsillar cancer patients with massive nodal burdens, the need to predict failure of locoregional or systemic control is increasing. Although disease control can be predicted by the tumor–node–metastasis (TNM) stage, it has some limitations in that it cannot represent the three-dimensional structure of tumors [10]. Furthermore, it is difficult to distinguish individual characteristics among tumors of the same size because it does not reflect the metabolism of tumors [11]. A recent study found that ^18^F-fluorodeoxyglucose (FDG) positron-emission tomography/computed tomography (PET/CT) parameters accurately predicted locoregional and systemic control; the maximum standardized uptake value (SUV_max_) correlated negatively with locoregional recurrence after definitive concurrent chemoradiation therapy [12]. Hoshikawa et al. used active monitoring (^18^F-FDG PET/CT) to calculate percentage changes in SUVs after chemoradiotherapy in an effort to predict disease control [13]. However, the data are insufficient to establish regular PET/CT as the standard imaging modality. Against this background, we analyzed the relationships between pretreatment ^18^F-FDG PET/CT parameters and distant metastasis in patients with tonsillar cancer. The purpose of this study was to evaluate the predictive value of ^18^F-FDG PET/CT parameters for disease control in tonsillar cancer.

## 2. Material & Methods

### 2.1. Patients

We retrospectively reviewed the medical records of 55 patients diagnosed with squamous cell carcinoma of the tonsils, who underwent pretreatment ^18^F-FDG PET/CT (cancer work-up) followed by primary surgery at Seoul St. Mary’s Hospital between January 2006 and January 2016. A single pathologist specializing in head and neck malignancies confirmed all cancers, and immunohistochemical markers including P16, P53, and Ki-67 were identified in pathological tissues obtained during surgery. The exclusion criteria were as follows: existing systemic metastasis; induction chemotherapy before surgery; previous treatment of head and neck cancer; an active malignancy in addition to head and neck cancer; clinical N0 stage according to preoperative imaging; and loss to follow-up. Clinical follow-up was performed monthly for the first 6 months, every 2 months during the following year, every 6 months in the next year, and once per year thereafter. The mean follow-up period was 52.33 months; the endpoints were overall and recurrence-free survival. All patients were staged according to the 8th edition of the American Joint Committee on Cancer staging system. This study was approved by our Institutional Review Board, which waived the need for informed patient consent given the retrospective nature of the work.

### 2.2. ^18^F-FDG PET/CT Protocol and Imaging Analysis

We instructed all patients to avoid excessive physical activity, and to fast for at least 6 h prior to ^18^F-FDG injection. We confirmed that the peripheral blood glucose level was <200 mg/dL prior to PET/CT. One hour after intravenous administration of 3.7–5.5 MBq/kg ^18^F-FDG, images were acquired using a combined PET/CT in-line system (Biograph Duo, Biograph TruePoint, Siemens Medical Solutions, Knoxville, TN, USA). The acquisition time was 2–3 min per bed position (six to seven positions for the whole body). All patients were in the supine position during the scan. Non-contrast-enhanced CT from orbitomeatal line to proximal thigh was performed using 80 mAs, 130 kVp, and 5 mm slice thickness (Biograph Duo) and 50 mAs, 120 kVp, and 5 mm slice thickness (Biograph TruePoint). PET scans of the same body region followed immediately. The CT data were used for attenuation correction, and PET data were reconstructed using a standard ordered subset expectation maximization algorithm.

PET/CT images were interpreted by two board-certified nuclear medicine specialists (H.L. Park and I.R. Yoo) with over 10 years of experience. All PET/CT scans were assessed using a single software (XD3, Mirada Medical, Oxford, UK). Semi-quantitative metabolic and volumetric parameters were measured by visually placing the region of interest around the site of primary tumors and metastatic lymph nodes. PET parameters were selected based on the literature, and included the SUV_max_ [14], SUV_peak_ [15], metabolic tumor volume (MTV), total lesion glycolysis (TLG), and tumor-to-liver uptake ratio (TLR). We fixed the SUV threshold at 2.5 to ensure that the entire region of interest (the MTV_2.5_) was within a contouring margin equal to or greater than an SUV of 2.5. The TLG was the sum of the tumor SUVs, calculated as MTV × SUV_mean_ [15,16]. The TLR was calculated as the ratio of tumor SUV max to mean SUV of the patient’s liver (3 cm^3^-sized volume of interest on the right hepatic lobe).

### 2.3. Surgery

**Figure 1:**
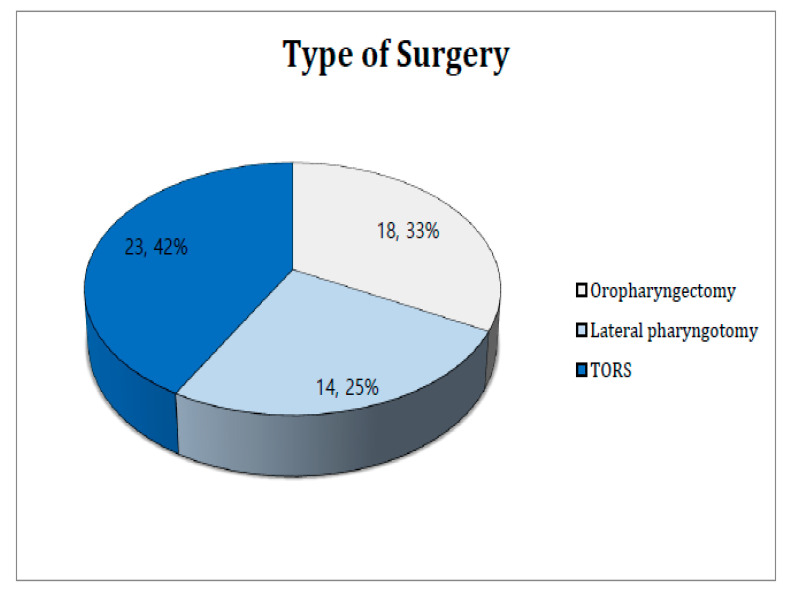
Type of surgery.

We performed oropharyngectomy using one of three approaches: conventional transoral lateral oropharyngectomy, transoral robotic surgery (TORS), or lateral pharyngotomy. Whether the Da-Vinci system was used for TORS depended on the tumor location and extent, and the provision of patient informed consent. As the Da-Vinci robotic surgery system was already installed prior to the study, TORS availability did not affect the choice of surgical approach. Figure 1 shows that TORS was preferred (23 cases; 42%), followed by conventional trans-oral lateral oropharyngectomy (18 cases; 33%) and lateral pharyngotomy (14 cases; 25%). The latter approach was chosen for patients with tumors beyond the tonsils (including the tongue base); such tumors were poorly exposed.

### 2.4. Statistical Analysis

Statistical analyses were performed using SPSS software (ver. 24.0; IBM Corp., Armonk, NY, USA). We calculated the mean and range for quantitative variables. Categorical variables were compared using the chi-squared and Fisher’s exact tests. Metabolic volumetric parameters were subjected to univariate analysis using a Cox’s proportional hazards regression model. The maximum Youden index (sensitivity + specificity − 1) was obtained by receiver operating characteristic (ROC) curve analysis and used to calculate the PET-CT parameter cutoffs. The Kaplan–Meier method was used to estimate 5-year overall survival rates and the groups were compared using the log-rank test. The 95% confidence intervals (CIs) were calculated for all parameters and the significance level was set to *p* = 0.05.

## 3. Results

### 3.1. Patients Characteristics

The patients were divided into two groups according to systemic metastasis status (Table 1). Of the 55 patients, there were 48 and 7 without and with metastasis, respectively. The lung was the most common metastatic organ (three patients; 33%), followed by bone and the brain/skull base (one patient each; 22%) and the small bowel and liver (one patient; 11%). The mean follow-up period was 52.33 months and the mean time to systemic metastasis was 31.71 months. Sex, smoking history, and HPV status did not differ significantly between the two groups. The TN stage was higher in the metastasis group, but not significantly. Pathological review revealed P53 immunohistochemistry in 20 (41.7%) patients without systemic metastasis and only 1 (14.3%) with metastasis (*p* = 0.054). Other pathological parameters, including the Ki-67 level, extranodal extension, perineural invasion, lymphovascular space invasion, and differentiation status did not differ significantly between the two groups. Concurrent chemoradiotherapy was the most common adjuvant treatment in both groups (26 of 48 patients [54.2%] in the systemic metastasis group and 4 of 7 [57.1%] in the group without metastasis); the rates did not differ significantly.

### 3.2. Metabolic Activity Parameters According to HPV Status

**Figure 2:**
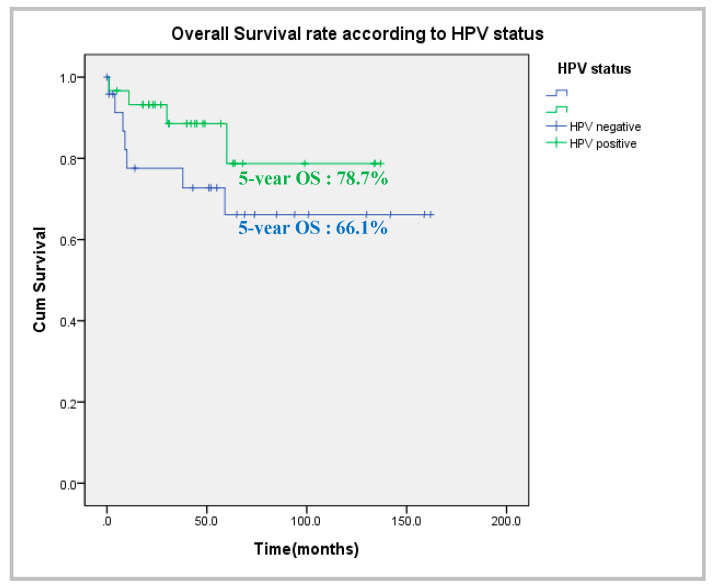
Comparison of overall survival according to HPV status.

The 5-year overall survival rate (Figure 2) was 78.7% in the HPV-positive group and 66.1% in the HPV-negative group. The univariate analyses of metabolic activity parameters are shown in Table 2 according to HPV status. The primary tumor SUV_max_ and SUV_peak_ did not differ between the two groups. However, the MTV_2.5_ and TLG_2.5_ of the HPV-negative group were 13.96 ± 13.49 cm^3^ and 89.15 ± 93.66, respectively, which were significantly higher than those of the HPV-positive group (8.99 ± 6.57 cm^3^ and 50.99 ± 43.99; *p* = 0.003 and *p* = 0.000, respectively). The metastatic lymph node MTV_2.5_ and TLG_2.5_ of the HPV-negative group were 13.80 ± 20.08 cm^3^ and 67.64 ± 104.51, respectively, which were significantly lower than those of the HPV-positive group (*p* = 0.034 and *p* = 0.029, respectively). There was no group difference when the MTV_2.5_ and TLG_2.5_ values of the primary tumors and metastatic lymph nodes were summed. The TLRs of the primary tumors and metastatic lymph nodes did not differ between the groups.

### 3.3. Metabolic Activity Parameters According to Systemic Metastasis Status

Table 3 shows the results univariate analysis of metabolic activity parameters according to systemic metastasis status. The primary tumor parameters did not differ by metastasis status. However, the metastatic lymph node MTV_2.5_, TLG_2.5_, and TLR of patients with systemic metastasis were 7.26 ± 3.03 cm^3^, 36.07 ± 54.24, and 183.46 ± 298.62, respectively, all of which were significantly higher than those of the patients without metastasis (18.76 ± 25.49 cm^3^, 88.94 ± 152.30, and 3.88 ± 1.62; *p* = 0.015, *p* = 0.041, and *p* = 0.051, respectively). When the primary tumor and metastatic lymph node values were summed, the MTV_2.5_ (286.55 ± 315.30 cm^3^) was the only parameter that was significantly higher in the systemic metastasis group (*p* = 0.036).

### 3.4. Cutoff Value, Area under the Curve (AUC), Sensitivity, and Specificity of MTV_2.5_

**Figure 3:**
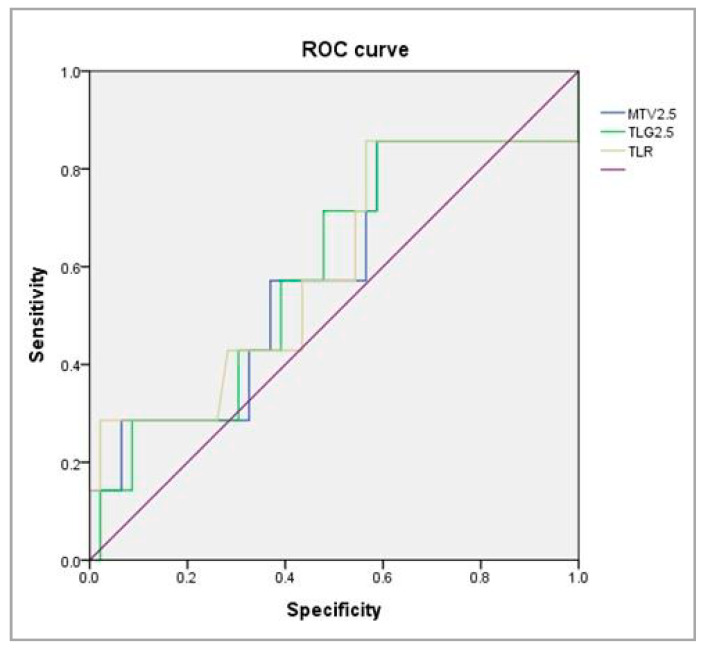
ROC curve analysis for searching the valuable parameters to predict systemic metastasis.

We constructed a ROC curve to calculate cutoff values. Figure 3 shows the AUC, sensitivity, and specificity of MTV_2.5_, TLG_2.5_, and TLR for predicting systemic metastasis. The MTV_2.5_ cutoff was ≥11.25 cm^3^, the AUC was 0.584, the sensitivity was 0.571, and the specificity was 0.565 (Table 4)

## 4. Discussion

Some reports of the relationships between the metabolic volumetric parameters of ^18^F-FDG and HPV-related cancers have appeared. Joo et al. showed that the SUV_max_ was significantly associated with negative findings for high-risk HPV (median cut off = 7.10) in oropharyngeal squamous cell carcinoma (OPSCC) patients [17]. Freihat et al. found no significant correlation between HPV status and ^18^F-FDG PET parameters including the SUV_max_, MTV, and TLG [18]. Thus, no consensus on the relationship between PET/CT parameters and HPV status has emerged. We found that some metabolic and volumetric parameters (the MTV_2.5_ and TLG_2.5_) in the HPV-positive group were lower for the primary tumors, but higher for metastatic lymph nodes, than in the HPV-negative group. As HPV-positive tonsillar cancer is associated with a greater risk of nodal metastasis from smaller primary tumors [19], the ^18^F-FDG PET parameters reflected a known behavior of tonsillar cancer.

Many studies have aimed to predict systemic metastasis, which is the major marker of disease control failure, in patients with head and neck cancer. T4 stage, current smoking, and cetuximab-based concurrent chemoradiotherapy were associated with significantly higher rates of distant metastasis and poorer prognoses [20]. Lymph node metastasis is also key for tumor dissemination in head and neck cancers. Cho et al. reported that lymph node metastases gave rise to more systemic metastases than primary tumors in an animal model, suggesting a role of the unique lymph node microenvironment in the formation of distant metastases [21]. The significance of lymph node metastasis for systemic dissemination has been also reported for other tumors. Recently, regional lymph node metastasis of breast cancer with lymphovascular invasion was found to be significantly correlated with systemic dissemination; lymphatic invasion allowed access to the systemic circulation [22]. The metastatic nodal burden of well-differentiated papillary thyroid cancer patients independently increased the risk of systemic metastasis, suggesting a need for individualized clinical treatment and surveillance according to the nodal stage [23]. Given the importance of the nodal burden in disease control, we investigated the relationship between the PET/CT metabolic parameters of metastatic nodes and systemic metastasis; higher nodal parameter values correlated with tumor dissemination. We found that the MTV_2.5_ of metastatic lymph nodes was positively associated with systemic metastasis. Similarly, Rich et al. reported that these parameters were useful for identifying cases likely to progress to distant metastasis after completion of HPV-positive OPSCC treatment [24]. Such findings may not be limited to tonsillar cancer. In patients with adenoidal cystic carcinoma of the salivary gland, all metabolic and volumetric parameters of PET/CT (including the SUV_max_, SUV_peak_, MTV, and TLG) significantly predicted distant metastasis; the risk increased 5.9-fold when the MTV exceeded 14.8 mL [25].

Traditionally, PET/CT SUV_max_ was used to predict metastasis of various malignant tumors, including cervical and lung cancer [26,27]. Especially for head and neck cancer, a primary tumor SUV_max_ > 9.0 negatively correlated with locoregional recurrence- and disease-free survival [28]. In a recent study, a pretreatment SUV_max_ > 6.0 of the largest metastatic lymph node predicted poorer regional recurrence-free survival of OPSCC patients [29]. However, given the heterogeneity of head and neck cancers, the SUV_max_ calculated using the highest voxel value of the entire tumor does not accurately reflect the tumor metabolic burden and is susceptible to imaging noise [30]. Several studies found that the SUV_max_ did not adequately predict the prognosis of patients with various tumors [31,32]. In a recent study, parameters such as the TLG and MTV, which measure overall tumor metabolic activity, were better correlated with the metastatic nodal burden and prognosis than the SUV, which is affected by many patients, technical, and physiological factors [33,34]. This supports our findings; the MTV and TLG results were significant.

The mechanism by which the metastatic nodal burden promotes tumor dissemination and disease progression remains incompletely understood; however, the immune tolerance induced by the upregulation of programmed cell death-1 ligand (PD-L1) expression may explain metastatic colonization [35]. Programmed cell death-1 (PD-1) is a checkpoint receptor expressed on T-cell surfaces that suppress the immune response to tumor cells [36]. A blockade of the interaction between PD-1 and PD-L1 in tumor and immune cells promotes an immune response to tumorigenesis that aids treatment of intractable tumors [37]. PD-L1 also promotes tumor cell glycolysis, thus exhausting the glucose required by T lymphocytes in tumor microenvironments [38]. The tumors therefore progress despite the immune response. We hypothesized that the tumor glycolysis promoted by PD-L1 might be revealed by PET/CT measurement of metabolic parameters. Indeed, Li et al. found that certain 18^F^-FDG PET parameters, especially the SUV_max_, MTV, and TLG, were significantly associated with PD-L1 levels > 1% and 50% (AUCs = 0.611 and 0.630, respectively) [39]. We drew a ROC curve to identify predictors of distant metastasis and their cutoffs. Of the three parameters investigated, only the MTV_2.5_ was useful (AUC = 0.584). We expect that preoperative PET/CT cancer work-up would be useful for predicting sensitivity to anti-PD-1/PD-L1 monoclonal antibody immunotherapy; combined with our results, this would provide more information for patients beginning treatment.

This study had some limitations. First, it was retrospective and performed in a single center. Second, the generalizability of the PET/CT metabolic and volumetric parameters is limited by the small sample size. Third, we did not analyze the predictive utility of the MTV_2.5_ according to HPV status because of the small number of HPV-positive patients. However, to the best of our knowledge, this is the first study to assess the predictive utility of PET/CT parameters for systemic metastasis and HPV status of tonsillar cancer patients. We expect that further research will lead to new tools to stratify patients according to risk and personalize clinical monitoring. Our results will aid prognostic predictions and individualized post-treatment surveillance; additional systemic therapy should be considered for patients at high risk of disease control failure.

## 5. Conclusions

We hypothesized that there may be a positive relationship between the nodal burden and distant metastasis of tonsillar cancer (regardless of HPV status), and that certain PET-CT metabolic and volumetric parameters might predict systemic dissemination. The MTV_2.5_ of metastatic lymph nodes, alone and summed with the primary tumor MTV_2.5_, was significantly correlated with systemic metastasis. Large-scaled, prospective study will be valuable to strengthen the predictive value of ^18^F-PET/CT parameters and our study could serve as a pioneer in the field of a reliable guideline in a multi-center trial.

## Figures and Tables

**Table 1:** Patient characteristics.

Parameters	Systemic Metastasis(-) (*n* = 48)	Systemic Metastasis(+) (*n* = 7)	*p* Value
Age (mean)	57.00 ± 7.81	60.86 ± 10.56	0.147
Sex (%)			0.205
Male	45 (93.8%)	6 (85.7%)	
Female	3 (6.2%)	1 (14.3%)	
Smoking (%)			0.101
Ex-smoker	20 (41.7%)	2 (28.6%)	
Current smoker	22 (45.8%)	4 (57.1%)	
Never smoker	6 (12.5%)	1 (14.3%)	
HPV status			0.337
Positive	25 (52.1%)	5 (71.4%)	
Negative	23 (47.9%)	2 (28.6%)	
TN stage			
Clinical T stage (%)			0.305
T1/T2	43 (89.6%)	6 (85.7%)	
T3/T4	5 (10.4%)	1 (14.3%)	
Clinical N stage at diagnosis (%)			0.120
N1/N2a	34 (70.8%)	4 (57.1%)	
N2b/N3	14 (29.2%)	3 (42.9%)	
Pathologic analysis			
p53 positive	20 (41.7%)	1 (14.3%)	0.054
Ki-67 (%)	67.37	76.67	0.241
Differentiation (well & moderately)	30 (62.5%)	5 (71.4%)	0.311
Lymphovascular space invasion	26 (54.2%)	5 (71.4%)	0.219
Perineural invasion	20 (41.7%)	3 (42.9%)	0.280
Extranodal extension	22 (45.8%)	4 (57.1%)	0.163
Adjuvant treatment modality (%)			
None	10 (20.8%)	1 (14.3%)	0.155
Radiation alone	12 (25.0%)	2 (28.6%)	
Chemotherapy alone	0 (0%)	0 (0%)	
Concurrent chemoradiotherapy	26 (54.2%)	4 (57.1%)	

**Table 2:** Univariate analysis of metabolic activity parameters according to HPV status.

Parameters	HPV Negative (*n* = 25)	HPV Positive (*n* = 30)	*p* Value
Primary tumor			
SUV_max_	11.09 ± 5.73	9.83 ± 3.60	0.157
SUV_peak_	8.94 ± 4.84	7.79 ± 3.28	0.166
MTV_2.5_	13.96 ± 13.49	8.99 ± 6.57	0.003
TLG_2.5_	89.15 ± 93.66	50.99 ± 43.90	0.000
TLR	5.53 ± 2.33	4.53 ± 1.59	0.220
Metastatic lymph node			
SUV_max_	7.856 ± 3.65	8.89 ± 3.40	0.389
SUV_peak_	6.133 ± 2.62	6.91 ± 3.02	0.701
MTV_2.5_	13.80 ± 20.08	19.45 ± 31.07	0.034
TLG_2.5_	67.64 ± 104.51	110.63 ± 204.50	0.029
TLR	4.05 ± 1.93	3.98 ± 1.73	0.369
Primary tumor + Metastatic lymph node			
SUV_max_	11.88 ± 5.18	10.99 ± 3.46	0.129
SUV_peak_	9.63 ± 4.38	8.74 ± 3.28	0.361
MTV_2.5_	27.20 ± 26.37	27.80 ± 33.56	20.391
TLG_2.5_	156.66 ± 144.78	160.66 ± 219.14	0.380
TLR	5.96 ± 2.04	5.08 ± 1.59	0.433

**Table 3:** Univariate analysis of metabolic activity parameters according to systemic metastasis.

Parameters	Systemic Metastasis(-) (*n* = 48)	Systemic Metastasis(+) (*n* = 7)	*p* Value
Primary tumor			
SUV_max_	10.14 ± 4.73	12.17 ± 4.24	0.147
SUV_peak_	8.08 ± 4.07	9.89 ± 4.01	0.167
MTV_2.5_	14.05 ± 12.94	17.46 ± 10.25	0.279
TLG_2.5_	73.92 ± 78.59	103.00 ± 72.62	0.156
TLR	4.84 ± 1.89	5.99 ± 2.61	0.232
Metastatic lymph node			
SUV_max_	8.18 ± 3.39	9.97 ± 4.29	0.260
SUV_peak_	6.45 ± 2.84	7.26 ± 3.03	0.254
MTV_2.5_	18.76 ± 25.49	36.07 ± 54.24	0.015
TLG_2.5_	88.94 ± 152.30	183.46 ± 298.62	0.041
TLR	3.88 ± 1.62	4.90 ± 2.77	0.051
Primary tumor + Metastatic lymph node			
SUV_max_	11.11 ± 4.39	13.14 ± 3.51	0.299
SUV_peak_	8.99 ± 3.84	10.19 ± 3.69	0.170
MTV_2.5_	32.02 ± 30.80	53.53 ± 57.78	0.036
TLG_2.5_	161.15 ± 173.03	286.55 ± 315.30	0.093
TLR	5.34 ± 1.74	6.42 ± 2.36	0.179

**Table 4:** Cutoff value, AUC, Sensitivity, and Specificity of MTV_2.5_ in the metastatic lymph node.

Cutoff Value	AUC (95% CI)	Sensitivity	Specificity
≥11.250	0.584 (0.336–0.832)	0.571	0.565

## Data Availability

The datasets used and/or analyzed during the current study available from the corresponding author on reasonable request.
